# Mesenchymal stem cells-derived exosomes for drug delivery

**DOI:** 10.1186/s13287-021-02629-7

**Published:** 2021-10-30

**Authors:** Yao Sun, Guoliang Liu, Kai Zhang, Qian Cao, Tongjun Liu, Jiannan Li

**Affiliations:** 1 Department of General Surgery, The Second Hospital of Jilin University, No. 218 Ziqiang Street, Changchun, 130041 China; 2 Operating Theater and Department of Anestheology, The Second Hospital of Jilin University, Changchun, 130041 China; 3 Department of Education, The Second Hospital of Jilin University, Changchun, 130041 China

**Keywords:** Mesenchymal stem cells, Exosomes, Drug delivery, Targeting

## Abstract

Exosomes are extracellular vesicles secreted by various cells, mainly composed of lipid bilayers without organelles. In recent years, an increasing number of researchers have focused on the use of exosomes for drug delivery. Targeted drug delivery in the body is a promising method for treating many refractory diseases such as tumors and Alzheimer's disease (AD). Finding a suitable drug delivery carrier in the body has become a popular research today. In various drug delivery studies, the exosomes secreted by mesenchymal stem cells (MSC-EXOs) have been broadly researched due to their immune properties, tumor-homing properties, and elastic properties. While MSC-EXOs have apparent advantages, some unresolved problems also exist. This article reviews the studies on MSC-EXOs for drug delivery, summarizes the characteristics of MSC-EXOs, and introduces the primary production and purification methods and drug loading methods to provide solutions for existing problems and suggestions for future studies.

## Introduction

Mesenchymal stem cells (MSCs) are multi-functional stem cells that are present in multiple human tissues. They can be found in the spinal cord, umbilical cord blood, umbilical cord tissue, placenta tissue, adipose tissue, etc. [1, 2]. With low immunogenicity, multi-directional differentiation ability, in particular homing ability, it has significant research potential in cardiovascular diseases, nervous diseases, and hematopoietic diseases [3]. The drug delivery system based on MSCs has become one of the most attractive therapeutic methods because of its characteristics. However, cells as drug carriers still face many problems such as uncertain differentiation accidents, cell embolism, infection, production, and storage [4]. As the secretion of MSCs, the exosomes inherit the relative advantages of MSCs and overcome the problems of MSCs as drug carriers [5].

The exosomes are secreted by mesenchymal stem cells (MSC-EXOs), most of which are cup-shaped or round with a diameter of 30–100 nm and a density of 1.13–1.19 g/ml [6]. The exosomes contain lipid, protein, and RNA [7]. The lipid bilayer maintains the integrity of exosomes and stabilizes biological activities. Protein modification on the surface enhances the recognition and targeting ability of the exosomes. Abundant RNA in the capsule improves the imaginative potential to regulate the transcription and translation of receptor cells. With different sources of MSC, the characteristics of the exosomes are different [8]. MSC-EXOs have many unique characteristics, such as small size, low immunogenicity, long-circulating half-life, good penetration, and good biocompatibility. It is one of the best choices for researchers to find drug carriers in vivo [9]. In current studies, researchers use MSC-EXOs as a carrier to deliver RNA, protein, and molecular drugs to specific parts of the body to achieve targeted therapy. However, problems such as carrier separation and purification, preservation and transportation, drug loading, and targeting still exist [10]. In this study, we first summarized the production and drug loading methods of MSC-EXOs, and the modification of MSC-EXOs for targeted drug delivery. Secondly, the delivery of RNA, small molecular drugs, and proteins by MSC-EXOs to treat different kinds of diseases was discussed. Furthermore, we also provided our perspectives for enhancing the drug loading efficacy of MSC-EXOs.

## MSC-EXOs

### Production of MSC-EXOs

As a drug carrier in vivo, a safe, efficient, and reliable purification method is necessary. The researchers improved the production of MSC-EXOs by changing the composition of MSC or the culture media [11].

MSCs cannot proliferate indefinitely, which limits the production of MSC-EXOs. The researchers transfected the MYC gene into MSC to obtain immortalized MSCs to increase the production of MSC-EXOs [12]. The results showed that MSCs transfected with the MYC grew faster, had less adhesion, and did not age. The exosomes produced by MSCs transfected with the MYC had the same effect as non-transfected MSCs. It showed that the MYC transfection is a feasible method for mass production of MSC-EXOs. The immortalization of MSC produces an infinitely expandable MSC clone cell line to increase the production of MSC-EXOs. This method allows MSC-EXOs to be produced from MSC of the same source, ensuring the consistency of different batches of MSC-EXOs as much as possible. It provided the possibility for the quality control of mass production of MSC-EXOs [13]. However, researchers indicated that the MYC is a proto-oncogene; it may cause unknown side effects in clinical practices [14].

Researchers have used scaffolds, spherical culture, hollow fiber bioreactor, and microcarrier-based 3D culture methods to achieve higher exosomes production [15]. Cao et al*.* inoculated MSCs into a hollow fiber bioreactor for 3D culture. The 3D culture increased the exosomes productions by 20 times compared with 2D culture. Transmission electron microscopy and western blotting were used to verify that there was no significant difference in the shape, size, surface markers of exosomes, and even better biological activity. It shows that it is feasible to use this method to increase yield [16]. Compared with the 2D culture system, the effective medium area of the 3D culture system is more extensive, and the distribution of exosomes in the supernatant is more concentrated, which improves the collection efficiency and reduces the use of consumables.

Stimulating MSC with biological materials or a specific culture media can also increase its paracrine capacity. Sung et al*.* made the comparison between thrombin, hypoxia, hydrogen peroxide, and lipopolysaccharide. The results showed that the four preconditions could enhance the production of exosomes. The thrombin could increase the yield of exosome by more than four times and enrich the exosome “goods” content [17]. However, these preconditions will produce unhealthy cells and excess MSC-EXOs [18]. 45S5 Bioglass® (BG) is one of the new generations of bioactive inorganic materials. The ions produced by BG can promote proliferation, differentiation, and secretion. They also provide a peaceful and friendly environment for the cells, so they will not be damaged. According to its characteristics, Wu et al*.* applied BG to the experiment of promoting MSC paracrine to produce more exosomes. The results showed that BG could stimulate MSC to produce more exosomes and enhance the biological function of exosomes by enhancing neutral sphingomyelinase-2 (nSMase2) and Rab GTPases pathways [19]. The exosomes production was increased by stimulating the MCS with the biological method. Compared with the traditional method of changing cell culture conditions, it has more advantages and lower costs.

From internal to external, from physical conditions to biomolecular regulation, researchers have proposed ways to increase the production of MSC-EXOs from various aspects. However, the methods proposed by the researchers will more or less affect the biological function of MSC-EXOs. And some of the impacts could be uncontrollable and unproven [11, 14]. In the future, researchers may need to integrate multiple methods to form standardized and consistent quality procedures to propose methods for mass production of MSC-EXOs.

### Separation and purification of MSC-EXOs

The exosome isolation and purification method will also affect the yield, purity, composition, and function of MSC-EXOs. At present, the widely used ultracentrifugation has limited scalability, fragmentation, and aggregation in terms of exosomes separation. Researchers developed new methods to optimize the separation and purification process of MSC-EXOs, including tangential flow filtration (TFF), precipitation method, and density gradient accumulation [20–25]. TFF is a method with solid expansibility and a high degree of purification. It can extract viruses and proteins from a large amount of cell culture fluid. Haraszti et al*.* combined TFF with 3D culture to obtain 20 times higher exosome yield than traditional methods [20]. However, TFF needs expensive equipment and is an open system with a higher risk of pollution. It still needs to be improved. Börger et al*.* referred to the polyethylene glycol (PEG) precipitation separation scheme of the virus. Considering the same physical characteristics of exosomes and viruses, PEG (10% PEG 6000) was applied to isolate MSC-EXOs. Results showed that the exosome yield was 14 times higher than that of traditional conventional differential centrifugation protocols [24]. The difference in the weight and concentration of PEG molecular would significantly influence the yield of exosomes but cannot affect the size of isolated exosomes. Ludwig et al*.* compared the yield of exosomes extracted of different molecular weights and concentrations of PEG. The results showed that 10–12% PEG 6000, 8–10% PEG 8000, and 8–10% PEG 20,000 were appropriate for separating exosomes. However, the dissolved PEG 20,000 was not suitable for MSC-EXOs isolation because its high viscosity makes it difficult to handle [26]. Studies have shown that densities influence the contents and characteristics of MSC-EXOs, so it is necessary to separate MSC-EXOs of different densities [27]. Gorgun et al*.* used density gradient centrifugation to separate vesicles of different sizes from mixed exosomes. A new flow cytometry method was also applied to characterize vesicles with a diameter of fewer than 1 μm [22]. Chopra et al*.* used ultracentrifugation (UC), sucrose cushion (SC), and total exosome isolation commercial reagent (CR) to separate Human Wharton's Jelly-Derived MSCs. The results showed that the exosomes extracted by SC have the highest purity [28].

So far, the various methods have limitations such as scalability, equipment complexity, purity control of the extracted vesicles, and pollution control. We should selectively integrate various methods and develop a complete set of separation and purification. This is a long way from large-scale therapeutical use [29].

### Quality control and safety

The drugs' massive production requires the following GMP (Good Manufacture Practice of Medical Products, GMP). As the production and purification process affects the properties of MSC-EXOs, it is necessary to set a standard for MSC-EXOs to achieve consistency of quality and safety [30]. Many studies have shown that MSC-EXOs transplanted into recipient organization are safe. Sun et al*.* conducted the intravenous injection on the rat model of myocardial infarction to evaluate the safety of transplantation hucMSC (human umbilical cord MSC). The experiment proved that the hucMSC is safe without any side effects [31]. However, this does not fulfill the quality and safety requirements in large-scale clinical applications. A report that “process is product” emphasizes quality control at every step and level. From the production of cells to purification, separation, storage, and final application, all are carried out following GMP standards [32]. Researchers should be highly aware of the administration dosage, stability, safety, toxicity, shelf life, and other biopharmaceutical characteristics.

### Loading methods

Efficient loading of biological cargo into MSC-EXOs is a very critical step. Currently, researchers used different loading methods for different types of biological cargo [33]. At present, methods such as electroporation, transfection, and overexpression are commonly used. The main advantages and disadvantages of the above three methods are shown in Table 1.

**Table 1:** The advantages and disadvantages of several commonly used loading methods

Methods	Type of cargo	Advantages	Disadvantages	References
Transfection	RNA	Good load efficiency and stability of the molecule	Potential toxicity and genetic changes	[36]
Electroporation	Hydrophilic cargo	Wide applicability	Require sophisticated equipment/possible molecular aggregation/morphological changes of exosomes	[37, 39–41]
Overexpression	Protein	Simple and convenient	May cause imbalance of cell proliferation and apoptosis	[33, 43]

Transfection is the most commonly used method to introduce RNA into exosomes. Lou et al*.* used plasmids to transfect miR-122 into adipose tissue-derived MSC (AMSC) successfully. The PCR method was used to check the expression level of miR-122 in exosomes [34]. There are also studies on directly transfecting RNA into exosomes, and good transfection efficiency has also been obtained. Zhang et al*.* reported a calcium chloride-mediated transfection method, which directly transfected the target RNA into exosomes instead of the parent cell [35]. However, studies have also pointed out that due to changes in gene expression and regulated transfection of production cells, an infection may cause potential toxicity or biosafety issues [36].

The electroporation technique uses an electric field to form temporary hydrophilic pores on the phospholipid membrane of the exosomes to load the biological cargo into the exosomes [37]. Gomari et al*.* used electroporation technology to load doxorubicin into the MSC-EXOs successfully. The loading efficiency was measured by a spectrophotometer [38]. However, some studies have pointed out that electroporation can cause RNA aggregation and changes in the morphology of exosomes [39]. RNA accumulation can cause the loaded RNA to lose its function, decreasing loading efficiency [40]. Hood et al*.* found that electroporation could increase the size of exosomes, forming aggregates. Such changes in the morphology of exosomes are not beneficial for maintaining the stability of exosome function in vivo and during storage. They developed a culture system containing trehalose, successfully maintaining the original size of exosomes and reducing the aggregation of exosomes [41].

Overexpression is often used for the loading of protein cargo. Transfection inserts gene fragments to regulate cell protein synthesis. The target protein will be loaded into the exosome through separation and purification. This method is technically mature and easy to operate. Huang et al*.* successfully loaded pigment epithelium-derived factor (PEDF) into ADSC-derived exosomes by overexpression and identified the expression of PEDF in exosomes by western blotting [42]. However, the report also pointed out that the overexpression of a specific protein may cause an imbalance of cell proliferation and apoptosis. This imbalance will reduce the proliferation rate of MSCs, inhibiting the production of exosomes. Exosomes may also be loaded with unwanted proteins with unpredictable effects on therapy. Therefore, more advanced sorting systems need to be developed to overcome the loading and expression of non-specific proteins [33, 43].

There are also other loading methods, such as incubation, ultrasonic treatment, and extrusion. However, there is a lack of horizontal comparison of different loading methods in the current research. For the clinical application of MSC-EXOs, it is necessary to find an optimal loading method.

### Targeting

In many diseases, targeted therapies are promising methods that reduce the adverse impact on other areas while improving the therapeutic effect of drugs on the target area. The researchers conducted a lot of studies on how to improve the targeting of MSC-EXOs.

#### Targeting peptide

Peptides conjugated on the surface of MSC-EXOs can improve the targeting of MSC-EXOs [44]. Using targeting peptides has been applied to research breast cancer, lung cancer, liver cancer, heart disease, and brain disease.

In order to achieve the targeting delivery of drugs to the acute myocardial infarction area, Wang et al*.* applied CTnI-targeted peptides on MSC-EXOs by gene-transfecting MSC cells. CTnI is highly expressed in the area of myocardial infarction, and the modified exosomes can be transported to this area through the concentration gradient of CTnI [45]. In the study of Wang et al*.,* they applied the IMTP (ischemic myocardium-targeting peptide) motif CSTSMLKAC onto MSC-EXOs membrane to target the ischemic myocardium. The results showed that IMTP exosomes significantly improved the targeting ability [46].

Exosomes can cross the blood–brain barrier, but recent studies have shown that exosomes accumulate in the liver and spleen after systemic injection, while the signal in the brain is relatively weak. Therefore, it is necessary to improve the distribution level of exosomes in the brain [47]. Cui et al*.* connected central nervous system-specific rabies viral glycoprotein (RVG) peptide on MSC-EXOs to achieve greater cortex and hippocampus targeting [48]. Jia et al*.* bound neuropilin-1-targeted peptide (RGERPPR, RGE) to the MSC-EXOs membrane by click chemistry to achieve targeting glioma. They also loaded superparamagnetic iron oxide nanoparticles (SPIONs) and curcumin (Cur) into MSC-EXOs. Their results showed that the modified MSC-EXOs can smoothly pass to the targeting area with good imaging and treatment effects [49].

Modifying the targeting peptide on the surface of exosomes is an efficient and direct method to improve the targeting of exosomes. This method requires selecting suitable targeting peptides for different lesions and choosing a suitable method to connect the targeting peptides with exosomes [50]. The main modification methods are genetic engineering, covalent modification, and non-covalent modification [51]. Adding targeting peptides can develop specific targeting peptides for each disease, and the application scenarios are extensive. However, challenges such as unexpected immunogenicity problems and the impact of the connection process still exist. These require researchers to better understand the characteristics of the surface composition, connection methods of MSC-EXOs, and the mechanism of disease.

#### Magnetic targeting

Studies have shown that an external magnetic field can control the biodistribution of iron oxide nanoparticles (IOPN) in the body after intravenous injection. The FDA (Food and Drug Administration) approves IOPN to treat iron deficiency anemia. It has biocompatibility and is ionized into iron ions and iron homeostasis. Thus, this is a feasible method to improve the targeting of exosomes. Kim et al*.* used IONP-harbored MSC to prepare the MSC-EXOs. The MSC-EXOs containing IOPN can navigate to the target area under the guidance of an external magnetic field. The results showed that the localization concentration increased by 5.1 times under in vitro magnetic navigation [52]. Kim et al*.* used PEGylated superparamagnetic IONP to improve the magnetization and colloidal stability in serum to prepare the IOPN-MSC-EXOs. The results showed that the magnetic navigation increased the amount of MSC-EXOs accumulating in the targeting area (spinal cord injury area) and the IOPN also stimulated the increase in therapeutic factors in the MSC-EXOs [53]. Lee et al*.* and Altanerova et al*.* also obtained similar results [54]. Jia et al*.* combined magnetic targeting and targeting peptides to enhance the targeting of MSC-EXOs. They loaded curcumin (Cur) and superparamagnetic IONP (SPIONs) into the MSC-EXOs and connected a membrane-targeting peptide. The RGE-Exo-SPION/Cur showed good targeting, biocompatibility, and therapeutic effects [49].

The magnetic navigation method is "non-invasive" compared to modifying the surface proteins of exosomes. It is an auspicious method with a lower impact on the exosomes.

#### Evaluation of targeting efficacy

As a promising drug for large-scale clinical use, it is inevitable to have precise tracking and biodistribution in vivo. Although researchers have conducted many experiments on MSC-EXOs, there is a lack of research on tracking and biodistribution in vivo [55]. At present, the primary methods for labeling MSC-EXOs in organisms are as follows: One is to wrap metal nanoparticles in exosomes, perform high-sensitivity magnetic resonance or CT analysis [52, 56], use radioactive tracers and nuclear imaging for tracking [57], and stained with fluorescent dyes [58]. Perets et al*.* developed a new visualization method that loaded glucose-coated GNPs (gold nanoparticles) into MSC-EXOs and used classical X-ray computed tomography (CT) to perform imaging to evaluate the biodistribution of MSC-EXOs in different pathological types of brain. The results clearly showed that the distribution of MSC-EXOs in the brain was closely related to inflammation [59]. Betzer et al*.* [60] and Cohen et al*.* [56] also used similar methods to evaluate the biodistribution of MSC-EXOs in the body. By studying the tracing of MSC-EXOs in the body, we can better research biodistribution, pharmacodynamics, targeting, etc.

The biodistribution of MSC-EXOs in the body is related to the source, size, injection method, and other factors of exosomes. MSC-EXOs accumulated more at the tumor site via local injection. They can also be quickly delivered into the liver and spleen via intravenous injection. Efficient exosomes delivery made the intranasal treatment more convenient.

## Delivery of different types of “drugs” in vivo

### Delivery of RNA by MSC-EXOs

#### Mechanism

RNA participates in the regulation of many cell activities. Among them, the most widely used MSC-EXOs targeted transport is miRNA. It can bind to the protein to change its function, target mitochondria to affect mitochondrial function, and activate the process of gene transcription. It can down-regulate the expression of noncoding genes and other processes to affect cell life activities. Therefore, the targeted transport of RNA is widely used in the research of various diseases [61]. By loading RNA, MSC-EXOs are often applied in the treatment of tumors, brain diseases, and spinal cord injuries.

Table 2 shows the study of MSC-EXOs RNA delivery in vivo in recent years [34, 62–86].

**Table 2:** Delivery of RNA by MSC-EXOs

RNA	Exosomes origin	Loading methods	Administration route	Model	Aim	References
miR-122	AMSC	Transfection	Intra-tumor injection	Mice	Enhance HCC chemosensitivity	[34]
miRNA-181-5p	AMSC	Transfection	Intrasplenic injection	Mice	Prevent liver fibrosis	[67]
microRNA-584	BMSCs	Transfection	Local injection	Mice	Reduce glioma activity	[62]
miR-132	BMSCs	Electroporation	Local injection	Mice	Promote angiogenesis	[107]
LNA-anti-miR-142-3p	BMSCs	Electroporation	Intravenous injection	Mice	Reduce tumorigenicity of breast cancer	[88]
miR-379	BMSCs	Transfection	Intravenous injection	Mice	Reduce breast cancer activity	[72]
miR-124	WJMSCs	Transfection	In vitro experiment	GBM cancer cells	Reduce glioblastoma multiform cells activity	[73]
miR-133b	BMSCs	Transfection	Intravenous injection	Mice	Neuroprotective role	[64]
miR-145-5p	HucMSCs	Transfection	Intra-tumor injection	Mice	Inhibit pancreatic ductal adenocarcinoma	[91]
miR-675	HucMSCs	Transfection	Local injection	Mice	Treat aging-induced vascular dysfunction	[110]
miR-126	BMSCs	Transfection	In vitro cell experiment	N/A	Ameliorate hypoxia/reoxygenation-injured ECs	[108]
miR-644-5p	BMSCs	Transfection	Intravenous injection	Cisplatin-induced POF mouse mode	Inhibit ovarian granulosa cell apoptosis	[74]
miR-34a	BMSC	Transfection	Intravenous injection	Mice	Reduce GBM activity	[93]
miR-29b	BMSC	Transfection	Intravenous injection	Mice	Repair spinal cord injury in rats	[63]
tsRNA-10277	BMSC	Transfection	In vitro cell experiment	N/A	Enhance osteogenic differentiation ability of dexamethasone-induced BMSCs	[75]
miR-126	BMSC	Transfection	Intravenous injection	Rats	Promote repair after spinal cord injury	[68]
MiR-29	BMSC	Transfection	Local injection	Rats	Potential treatments for AD	[96]
miR-499	BMSC	Electroporation	Intratumorally injected	Mice	Inhibit endometrial cancer growth and metastasis	[76]
miR-544	BMSCs	Transfection (incubate together)	Intravenous injection	Rats	Promote repair after spinal cord injury	[97]
miR-199a	AMSCs	Transfection	Intravenous injection	Mice	Improve HCC chemosensitivity	[66]
miR-29a	BMSCs	Transfection	Intravenous injection	Mice	Promote angiogenesis and osteogenesis	[112]
LNA-antimiR-142-3p	BMSCs	Electroporation	In vitro cell experiment	Mice	Reduce the tumorigenicity of breast cancer stem cells in vivo	[89]
miRNA-126-3p	HucMSCs	Transfection	In vitro cell experiment and intravenous injection	Mice	Enhance endothelial function and attenuate vein graft neointimal formation	[77]
miR-326	HucMSC	Transfection	Intravenous injection	Mice	Relieve DSS-induced IBD	[78]
miR-19a/19b	BMSCs	Transfection	Intravenous injection	Mice	Mitigate the damage of myocardial infarction	[70]
MiR-17–92	BMSCs	Transfection	Intravenous injection	Mice	Enhance axon-myelin remodeling and motor electrophysiological recovery after stroke	[65]
miR-124	BMSCs	Transfection	Subcutaneous injection	Mice	Treatment of pancreatic tumors	[71]
miR-124	HADMSCs	Transfection	In vitro cell experiment	M17 human NB cell line	Therapy for NB cancer	[79]
miR-214	BMSCs	Transfection	Intracerebroventricular injection	Rats	Neuroprotection against cerebral	[80]
miR-381	ADMSC	Electroporation	In vitro cell experiment	MDA-MB-231 cells	Inhibit triple-negative breast cancer aggressiveness	[81]
aiR-34a	Dental pulp MSCs (DPSCs)	Transfection (Lentiviral miR-34a transduction)	In vitro cell experiment	MDA-MB-231 cells	Repress proliferation of breast carcinoma cells	[82]
miR-135B	BMSCs	Transfection	Articular cavity injection	OA rat model	Attenuate the damage of cartilage tissues	[83]
miR-155-5P	Synovial MSCs (SMSCs)	Transfection	Articular cavity injection	OA rat model	OA therapy	[84]
miR-126	BMSCs	Transfection	In vitro cell experiment	Human umbilical vein endothelial cells	Pro-angiogenic potential	[85]
MicroRNA-let7c	BMSCs	Lentiviral transduction	Administered intravenously	Rats	Attenuate renal fibrosis	[86]
PTEN-siRNA	BMSCs	Incubated	Intranasal injection	Rats	Promote recovery for SCI	[98]
miR-21	HucMSC	Transfection	In vitro cell experiment	β-cells	Treatment of diabetes	[100]
miR-375	BMSCs	Transfection	In vitro cell experiment	β-cells	Treatment of diabetes	[101]
miR-125a	ADMSC	Transfection	Intravenous injection	Rats	Diabetic nephropathy	[104]
miR-126	HucMSC	Transfection	Local injection	Rats	Diabetic eye diseases	[105]
lncRNA H19	BMSCs	Transfection	Local injection	Rats	Diabetic foot ulcer	[106]

N/A, not applicable

#### For Tumor

Many studies have shown that the occurrence, proliferation, and metastasis of tumors are closely related to the abnormal levels of specific RNAs [69]. Researchers tried to use MSC-EXOs' targeting ability to regulate the level of specific RNA to treat tumors. Currently, RNA targeting has been expressively applied to breast cancer, hepatocellular carcinoma, and pancreatic ductal adenocarcinoma (PDAC). Breast cancer is the highest incidence of malignant tumors in women. Because of its late metastasis, it can cause multiple organ diseases, which seriously threaten the lives of patients. Studies have shown that the up-regulation of miR-142-3p and miR-150 is related to the tumorigenic breast cancer stem cells [87]. Therefore, Naseri et al*.* loaded LNA (locked nucleic acid)-modified anti-miR-142-3p oligonucleotides into MSC-EXOs. They used MSC-EXOs to deliver anti-miR-142-3p to breast cancer sites to enforce the expression of miR-142-3p and miR-150 in cancer cells. The results showed that, whether in vivo or in vitro, MSC-EXOs can deliver LNA-anti-miR-142-3p to the target site, enforce the expression of target RNA, and improve the transcription level of target genes APC and P2X7R. The results also showed that the MSC-EXOs had good biodistribution and successfully suppressed tumor growth [88, 89]. Hepatocellular carcinoma is highly resistant to conventional chemotherapy. Studies have shown that microRNA-122 (miR-122) is closely related to the occurrence and development of hepatocellular carcinoma and chemotherapy sensitivity [90]. Therefore, Lou et al*.* used MSC-EXOs to deliver miR-122 to the tumor tissue to improve the sensitivity of hepatocellular carcinoma to chemotherapy. The results showed that MSC-EXOs successfully delivered miR-122 and increased the sensitivity of hepatocellular carcinoma to sorafenib. ADAM10, IGF1R, and CCNG1 may be the predicted targets of miR-122, and down-regulating their expression levels can induce cell apoptosis and cell cycle arrest, thereby improving the chemotherapy sensitivity of hepatoma [34]. Lou et al*.* used MSC-EXOs to deliver miR-199a to target the mTOR (an oncogene related to cancer chemotherapy sensitivity) pathway, which also successfully improved the sensitivity of hepatocellular carcinoma [66]. PDAC is one of the cancers with a very high mortality rate. The lack of effective treatments makes it an incurable disease for most patients. Ding et al*.* used MSC-EXOs to deliver exogenous miR-145-5p. The results showed that it successfully inhibited the proliferation and metastasis of cancer cells in mice. The author believed that this may be closely related to miR-145-5p targeting mRNA 3'UTR regions of Smad3 (Recombinant Human Mothers Against Decapentaplegic Homolog 3) to down-regulate its expression [91].

The treatment of cancer by MSC-EXOs can start from the following aspects. On the one hand, the close relationship between RNA and cell activities can directly inhibit the growth of cancer cells. On the other hand, RNA can be delivered to improve the sensitivity of cancer cells to radiotherapy and chemotherapy to optimize the treatment effect. The RNA changes in cancer are diverse with increase and decrease. In future research, we may treat multiple RNA changes simultaneously to get more reliable results.

#### For brain diseases

Central nervous system diseases are hard to treat due to limited surgical treatment options. The fragile brain tissue and low penetration through the blood–brain barrier result in inadequate treatment and prognosis for many diseases. With the emergence of MSC-EXOs, researchers have discovered that using MSC-EXOs to carry RNA molecules to reach the central nervous system is a promising method for treating related diseases [62]. Glioma (GBM) is a malignant tumor with an inferior prognosis in the brain. Studies have shown that the occurrence and development of GBM are closely related to the abnormal activity of individual miRNAs [92]. Wang et al*.* found that the expression of miR-34a is down-regulated in GBM cells, while the expression of MYCN (a member of the MYC family that encodes a protein with a primary helix–loop–helix domain) is up-regulated. MiR-34a can target and negatively regulate the expression of MYCN. They loaded miR-34a into MSC-EXOs and performed in vivo and in vitro experiments. The results showed that the growth and proliferation of GBM were successfully inhibited, and the chemotherapeutic sensitivity of GBM cells to Temozolomide (TMZ) was improved by silencing MYCN [93]. Glioblastoma is the most common malignant tumor in the adult brain. Due to its strong invasive ability, the survival time of patients is short, and the surgical effect is little [94]. Therefore, by exploring its pathogenesis, it has great potential to use the drug transportability of MSC-EXOs to find its treatment.

AD is a progressive neurodegenerative disease with insidious onset. The exact cause and effective treatment are still unclear. Studies have found that MiR-29 is down-regulated in AD, which may be related to the pathogenesis of AD [95]. Jahangard et al*.* loaded MiR-29 in MSC-EXOs and delivered it to the hippocampus by local injection. The target genes of MiR-29 are mainly BACE1 (β-site amyloid precursor protein cleaving enzyme 1) and BIM (Bcl-2 interacting mediator of cell death (BCL2-like11)). The results showed that miR-29 was up-regulated, with its main targets down-regulated in hippocampus areas injected with MSC-EXOs. They reduced the pathological effects of amyloid-β (Aβ) peptides in a rat model of AD. This study proved that engineered exosomes had a specific protective effect on amyloid pathogenesis and showed a positive correlation in restoring the learning function of the hippocampus. However, there is a lack of data on the biodistribution of MSC-EXOs and the ability of MSC-EXOs to cross the blood–brain barrier successfully. The data are essential for the clinical application of the drug and need to be explored further [96]. In this study, the local injection was used to inject exosomes into the hippocampus. In clinical applications, local injection is highly effective in some cases but is not suitable when the drug has high concentration, strong irritation or large single injection volume. For AD, it is still essential to explore its exact pathogenesis. Once its mechanism is clarified, the related drug delivery methods will positively affect AD treatment.

#### For spinal cord injury

Spinal cord injury (SCI) is a severe neurological disease that can cause sensory and motor nerve dysfunction in corresponding spinal cord segments. Due to the poor regeneration function of neurons, there is still a lack of clinically effective treatment methods so far. The development of stem cell transplantation has brought a new direction for the treatment of SCI, but because stem cell transplantation faces many risks, researchers have found that using MSC-EXOs to deliver related RNA can also achieve neuron protection or repair. The researchers loaded miRNA-29b, miR-126, and miR-544 into MSC-EXOs by transfecting MSC [63, 68, 97]. The results show that the above three studies have achieved good results. Exosomes carrying the corresponding RNA promoted angiogenesis and nerve regeneration after spinal cord injury, and spinal cord injury has been significantly improved. The above three studies all achieved drug delivery through intravenous injection, and some researchers creatively used intranasal injection to treat spinal cord injury. Guo et al*.* loaded phosphatase and tensin homolog small interfering RNA (ExoPTEN) into MSC-EXOs to reduce the expression of PTEN in the area of spinal cord injury. PTEN is expressed in neurons and regenerating axons and can inhibit the growth of axons by down-regulating cytoplasmic mammalian target of rapamycin (mTOR) activity. The results showed that after intranasal administration, MSC-EXOs successfully migrated to the injury site to improve neurological function. It has a better effect on the injury site compared to intravenous injection [98]. So far, the mechanism of using MSC-EXOs to deliver RNA to treat spinal cord injury is still unclear. Further researches need to be conducted to see if there is any other treatment method for different impacts.

In general, the use of MSC-EXOs for the targeted delivery of RNA is effective. MSC-EXOs from different sources contain different kinds of abundant RNA molecules. Researchers adjust the cell activities by changing the level of the corresponding RNA or loading exogenous RNA molecules to treat certain diseases. Both in vivo and in vitro experiments have promising results. However, the current research lacks evidence on whether RNA loading into normal cells will adversely affect it. Researchers need to further explore the operating mechanism of RNA involved in cell activities and improve the MSC-EXOs targeting to reduce the impact on normal cells.

#### For diabetes and its complications

Diabetes is a metabolic disease characterized by chronic hyperglycemia. β-cells play an important role in both type 1 and type 2 diabetes. Therefore, reversing β cell injury or promoting β-cells regeneration is crucial in diabetes treatment [99]. Chen et al*.* explored the effect of miR-21 loaded with MSC-EXOs on the apoptosis and damage of β-cells caused by hypoxia. The results showed that miR-21 could alleviate endoplasmic reticulum stress and inhibit p38 MAPK signaling to protect β-cells against apoptosis induced by hypoxia [100]. Wen et al*.* used MSC-EXOs to transport siFas and anti-miR-375 to the islets. The results showed that it could down-regulate Fas and miR-375 levels, suppress β-cells apoptosis, and improve the viability and function of human islets against inflammatory cytokines [101]. Islet transplantation is the most effective diabetes treatment. β-cells apoptosis caused by hypoxia and immune rejection after transplantation are the main obstacles to islet transplantation [100, 101]. The above two experiments overcame the above obstacles by MSC-EXOs carrying RNA, providing a new solution for the successful islet transplantation.

Long-term hyperglycemia can potentially lead to microvascular disease and multiple serious tissue and organs complications, such as diabetic nephropathy, diabetic eye disease, and diabetic foot ulcer (DFU) [99, 102]. Diabetic nephropathy is related to genetic and environmental factors, causing continuous and irreversible damage to the glomeruli and tubulointerstitium of the kidney [103]. Hao et al*.* transfected miR-125a mimic into MSC-EXOs. The results indicated that miR-125a carried by MSC-EXOs could bind to histone deacetylase 1, thus down-regulating the endothelin-1 expression and reducing the diabetic nephropathy-like symptoms and renal impairment. Therefore, the development of diabetic nephropathy could be delayed [104]. Diabetic retinopathy is one of the main manifestations of diabetic eye disease. Zhang et al*.* found that MSC-EXOs overexpressing miR-126 could down-regulate the high-mobility group box 1 signaling pathway to inhibit the activation of NF-jB/p65 (a mediator that regulated the expression of inflammatory factors), thus improving hyperglycemia-induced retinal inflammation [105]. Long noncoding RNA (lncRNA) H19 was transfected into MSC-EXOs to explore its role in the healing process of a mouse with DFU. The results showed that the lncRNA H19 in MSC-EXOs could inhibit microRNA-152-3p and promote phosphatase and tensin homolog expression, thus regulating the proliferation and migration of fibroblasts and promoting the recovery of DUF. The blocked phosphatidylinositol-4,5-bisphosphate 3-kinase (PI3K)/protein kinase B (Akt) signaling pathway also can promote DUF recovery [106]. Controlling blood sugar levels is essential to treat the complications of diabetes. Diabetes complications are mostly related to the microvascular disease under high glucose levels in the body, but the specific mechanism is still unclear and needs to be further explored [99]. At present, diabetes and its complications still lack effective radical cure methods. MSC-EXOs is expected to become a new carrier to treat diabetes and its complications due to its rich internal regulatory factors and special biological characteristics.

#### For cardiovascular disorders

In cardiovascular diseases, MSC-EXOs can be applied to transport RNA to the lesion site, which can regulate angiogenesis, promote heart function recovery, and inhibit myocardial fibrosis. Ma et al*.* used electroporation to load miR-132 into MSC-EXOs to explore its effect on angiogenesis. The results showed that miR-132 in MSC-EXOs targeted RAS p21 protein activator 1 (a negative regulator of blood vessel germination and blood vessel branching) and increased tube formation of endothelial cells in in vitro experiments. In the mouse myocardial infarction model, MSC-EXOs promoted the formation of new blood vessels in the infarcted area and the recovery of heart function [107]. Similarly, Pan et al*.* delivered miR-126 mimic to endothelial cells through MSC-EXOs, which activated the PI3K/Akt/eNOS pathway, down-regulated cleaved caspase-3 expression, and increased the expression of pro-angiogenic factors, thereby enhancing survival and angiogenesis in endothelial cells damaged by hypoxia/reoxygenation [108]. According to the above studies, MSC-EXOs can be used as a natural therapeutic delivery vehicle, absorbed by endothelial cells. MSC-EXOs play an important role in preventing endothelial cell damage caused by hypoxia/reoxygenation and promoting angiogenesis and revascularization. In addition to promoting angiogenesis, MSC-EXOs-loaded RNA can inhibit myocardial fibrosis in myocardial infarction. Wang et al*.* loaded miR-19a/19b into MSC-EXO by transfection and introduced it into the infarcted area, while transplanting MSCs to explore the impact on myocardial infarction. The results showed that the MSC-EXO inhibited the apoptosis of cardiac HL-1 cells and reduced the area of fibrosis of cardiac tissue in a mouse myocardial infarction model. Interestingly, the reduction in myocardial fibrosis did not prevent the scar formation (an important process to maintain the structural integrity of the ventricular wall), but only reduced the harmful effects of myocardial fibrosis [70]. The research creatively transplanted both MSC-EXOs and MSCs into the infarcted area, promoting the recovery of heart function and reducing myocardial fibrosis. This study provided a new “combination” form in the field of cardiac protection, but extensive experiments are needed to verify the biosafety of this strategy.

#### For senescence-related disorders

Aging can cause organ system diseases such as vascular dysfunction and osteoporosis. However, its specific mechanism is unclear yet, and effective prevention methods are not available. Oxidative stress is closely related to the pathogenesis of aging-related diseases, which activates the transforming growth factor-β (TGF-β) pathway and subsequently up-regulates senescence markers p21 [109, 110]. Han et al*.* loaded miR-675 into MSC-EXOs by transfection to explore its therapeutic effect on senescence-induced vascular dysfunction. According to the results, miR-675 can inhibit the expression of TGF-β1 protein by targeting TGF-β1 and then down-regulate the TGF-β1/pSMAD/p21 signaling pathway, thereby preventing aging-induced vascular dysfunction. In the study, MSC-EXOs were encapsulated in a hydrogel to improve the stability and half-life of exosomes and the therapeutic effect of exosomes [110]. Angiogenesis is closely related to the formation and regeneration of bones [111]. Lu et al*.* reported that MSC-EXOs loaded with miR-29a can promote angiogenesis and osteogenesis. They found that miR-29a in old bone marrow mesenchymal stem cells (BMSCs) lines is significantly lower than that in young ones, which may be related to bone loss in old age. MSC-EXOs loaded with miR-29a can promote bone mass increase by promoting blood vessels formation, which may become a potential treatment for senescence-related osteoporosis [112]. The proliferation and differentiation ability of BMSCs also play an important role in bone formation. Jia et al*.* explored the effect of young MSC-EXOs on old BMSCs, and the results showed that MSC-EXOs enhanced the proliferation and osteogenic ability of BMSCs [113]. However, the experiment did not explain which components in MSC-EXOs work. We can further explore the ingredients that play a role in MSC-EXOs and use engineering technology to increase the content of corresponding components. Then, using MSC-EXOs to transport ingredients can become a new solution for bone diseases in elderly patients. In general, further exploration of the pathogenesis of aging-related diseases is the key.

### Delivery of small molecule drugs by MSC-EXOs

Targeted delivery can improve the efficacy of the drug on the target tissue and reduce the side effects of the drug. Researchers have loaded doxorubicin, paclitaxel, magnolol into MSC-EXOs to achieve targeted treatment of various diseases. Targeted delivery of MSC-EXOs is primarily used in the treatment of tumor. Gomari et al*.* explored the use of MSC-EXOs to deliver paclitaxel and adriamycin to treat breast cancer. They first compared the delivery efficiency of targeted modified MSC-EXOs loaded with doxorubicin and unmodified MSC-EXOs and free doxorubicin on HER2-positive breast cancer cells. The results showed that the delivery efficiency of targeted modified MSC-EXOs was significantly higher than that of the control group, but this difference gradually decreased as the concentration of MSC-EXOs increased. This result indicates that doxorubicin can be delivered through the targeted modified MSC-EXOs to achieve a higher level of targeted delivery at a lower systemic dose level, reducing its systemic side effects [38]. Then, Gomari et al*.* demonstrated that doxorubicin delivered by MSC-EXOs could significantly reduce the growth rate of tumors in a mouse breast cancer model [114]. Melzer et al*.* loaded paclitaxel into MSC-EXOs to explore its anti-tumor effect. The results showed that MSC-EXOs loaded with paclitaxel significantly reduced the tumor volume and inhibited the distant metastasis in the liver, spleen, and kidneys. More importantly, the use of paclitaxel was also significantly reduced [115]. In addition to breast cancer, MSC-EXOs loaded with anti-cancer drugs have also been used to treat other cancers, such as liver cancer, pancreatic cancer, and oral squamous cell carcinoma (Table 3) [116–120]. At present, because most anti-cancer drugs damage normal tissues and have strong side effects, loading drugs into MSC-EXOs for targeted delivery in vivo is mainly used to treat malignant tumors. Loading small molecule drugs into MSC-EXOs to treat inflammation, tissue regeneration, and other treatments is also very promising. Current research results show that small molecule anticancer drugs can be successfully loaded into MSC-EXOs to deliver to the target site with higher efficiency, maintaining a good drug release curve. The homing performance and specific ligands connection of MSC-EXOs also improved its targeting ability. However, the mechanism of the tumor is complicated. Some types of exosomes can even promote tumor proliferation. Therefore, it is necessary to choose a suitable source for MSC-EXOs to load with, and more researches need to be conducted on the relationship between exosomes and different tumors.

**Table 3:** Delivery of DRUG by MSC-EXOs

Aim	Exosomes origin	Loading methods	Administration route	Model	DRUG	References
Breast cancers	HucMSC	Incubation	Intravenous injection	Mice	Paclitaxel	[115]
Breast cancers	MSC	Electroporation	In vitro cell experiment		Doxorubicin	[114]
Hepatocellular carcinoma	BMSC	Electroporation	Intravenous injection	Mice	Norcantharidin (NCTD)	[117]
Osteosarcoma	BMSC	Dialyze	In vitro cell experiment		Doxorubicin	[119]
Pancreatic cancer	BMSC	Electroporation	Intravenous injection	Mice	Gemcitabine monophosphate(GEMP)	[120]
Oral squamous cell carcinoma	MSC	Incubation	Injected subcutaneously	Mice	Cabazitaxel (CTX)	[116]
Several cancer cell lines	MSC	Sonication	In vitro cell experiment		Honokiol	[118]

### Delivery of protein by MSC-EXOs

Protein is the primary component of life structure, and it plays an essential role in various life activities in the body. Researchers have loaded growth regulators, tumor necrosis factors, enzymes, and other proteins into MSC-EXOs. Explore the targeted delivery potential of MSC-EXOs-loaded proteins. It is mainly used in the treatment of brain injury, cardiovascular disorders, tumor, etc. Huang et al*.* increased the content of PEDF in adipose-derived MSCs (ADSCs) by overexpression to identify its protective effect on brain injury through in vitro cell experiments and in vivo experiments. The results showed that MSC-EXOs modified by PEDF successfully ameliorated the cerebral ischemia–reperfusion injury by regulating cell autophagy and apoptosis. However, this study did not explore the penetration of the blood–brain barrier and the distribution of MSC-EXOs carrying PEDF in vivo [42]. MA et al*.* overexpressed Akt into the human umbilical cord (hucMSCs) to study how it works in myocardial infarction. The results show that it accelerates the endothelial cell proliferation and migration and blood vessels formation in vitro and significantly improves heart function in vivo experiments. The researchers pointed out that this may be related to the up-regulation of platelet-derived growth factor D (PDGF-D) expression by AKT-MSC-EXOs [121]. Liu et al*.* delivered angiopoietin-2 (Ang-2) to human umbilical vein endothelial cells (HUVECs) through MSC-EXOs, which enhanced the proliferation, migratory, and tube-forming ability of HUVECs [122]. Zhuang et al*.* used genetic engineering methods to anchor cell-penetrating peptides (CPPs) and tumor necrosis factor (TNF-α) on the surface of MSC-EXOs. SPIONs are connected to the surface through chemical reactions. The results showed that CTNF-α-exosome-SPIONs obtained better anti-tumor properties and reduced the side effects in vivo. The research creatively anchored the “cargo” on the surface of exosomes instead of inside them, providing a new idea for the targeted transportation of proteins in the future [123].

At present, the primary method of loading protein into MSC-EXOs is to overexpress the protein. Whether this method of adjusting the protein content will affect other functions of the cell is temporarily unknown. Studies have also pointed out that exosomes are most likely to act through protein rather than RNA [124]. Therefore, further exploring the mechanism of RNA and protein in exosomes is necessary. Other studies also confirmed that MSC-EXOs are feasible as a protein transport carrier [122, 125–128] (Table 4).

**Table 4:** Delivery of protein by MSC-EXOs

Aim	Exosomes origin	Loading methods	Administration route	Model	Protein	References
Ameliorate cerebral ischemia–reperfusion injury	ADSCs	Overexpression	Local injection	Mice	PEDF	[42]
Improve cardiac regeneration	HucMSCs	Transfection	Intravenous injection	Mice	Akt	[121]
Anti-tumor	MSC	Electroporation	In vitro cell experiment	N/A	TNF-α	[123]
Anti-tumor	BMSCs	Transfection	Intravenous injection	Mice	TRAIL①	[126]
Cystic fibrosis	BMSCs	Transfection	In vitro cell experiment	N/A	Zinc Finger Protein	[128]
Prevent cisplatin injury	HucMSC	Transfection	In vitro cell experiment	N/A	14–3-3protein	[125, 127]
Enhance angiogenesis	HucMSCs	Overexpression	Subcutaneous injection	Mice	Angiopoietin-2	[122]

N/A, not applicable. ①TRAIL, tumor necrosis factor-related apoptosis-inducing ligand

## Conclusions and perspectives

According to recent studies, both advantages and challenges are identified in the area of MSC-EXOs delivery. The summary below includes the advantages and challenges of MSC-EXOs as drug delivery system:

Advantages: (1) Many studies have shown that the biological function of MSCs is achieved by paracrine EV. MSC-EXOs perfectly inherit the advantages of low immunogenicity and solved many problems in drug delivery. (2) MSC-EXOs contain a wide variety of proteins, RNA, and other molecules. Increasing or reducing the content of a specific molecule can easily reach the regulation of a specific disease pathway. (3) The surface and interior of MSC-EXOs can be modified, and various targeting molecules can be attached to its surface to improve MSC-EXOs targeting, which has robust expandability.

Challenges: (1) At present, researchers have proposed a variety of optimization methods for MSC-EXOs production and purification, but most methods lack standardization and quality control. The contents and functions of MSC-EXOs are different. Before the standardized production of MSC-EXOs, the accurate judgment and selection of MSC-EXOs are necessary. Integrating standardized, convenient, and strictly controlled production and purification methods is a promising solution. (2) The improvement in MSC-EXOs targeting is highly dependent on its targeted modification, which requires a better understanding of the mechanism of disease occurrence and development. For unclear pathogenesis such as Parkinson's and Alzheimer's, it is necessary to find more efficient and accurate targeting molecules or methods by exploring its pathogenesis to improve drug delivery targeting. (3) The biodistribution and half-life of MSC-EXOs in the human body need to be further clarified. The main routes of administration of MSC-EXOs are intravenous injection, local injection, intranasal administration, etc. The different routes of administration will affect the biodistribution of the drug in the body, which requires further comparison and exploration by researchers. The detection of the biodistribution of MSC-EXOs in vivo is closely related to the in vivo labeling of MSC-EXOs. Researchers can combine imaging and other methods with the labeling technology of MSC-EXOs to explore its biodistribution in the body. (4) Current disease models are mostly mice or in vitro cell experiments used for cancer and other disease studies, which are relatively simple and lack data for comparative research with other disease models.

Accurate drug delivery in the human body can improve drug efficacy and reduce side effects. MSC-EXOs, as one of the most potential in vivo transport carriers, need to be further studied and developed by researchers.

## Data Availability

All data generated or analyzed during this study are included in this published article.

## Abbreviations

MSC-EXOs: The exosomes secreted by mesenchymal stem cells
MSCs: Mesenchymal stem cells
BG: 45S5 Bioglass®
nSMase2: Neutral sphingomyelinase-2
TFF: Tangential flow filtration
UC: Ultracentrifugation
SC: Sucrose cushion
CR: Commercial reagent
GMP: Good Manufacture Practice of Medical Products
HucMSC: Human umbilical cord MSC
AMSC: Adipose tissue-derived MSC
IMTP: Ischemic myocardium-targeting peptide
RVG: Rabies viral glycoprotein
SPIONs: Superparamagnetic iron oxide nanoparticles
Cur: Curcumin
IOPN: Iron oxide nanoparticles
FDA: Food and Drug Administration
GNPs: Gold nanoparticles
PDAC: Pancreatic ductal adenocarcinoma
LNA: Locked nucleic acid
miR-122: MicroRNA-122
GBM: Glioma
MYCN: A member of the MYC family that encodes a protein with a primary helix–loop–helix domain
TMZ: Temozolomide
AD: Alzheimer's disease
SCI: Spinal cord injury
PEDF: Pigment epithelium-derived factor
ADSCs: Adipose-derived MSCs
Akt: Protein kinase B
PDGF-D: Platelet-derived growth factor D
CPPs: Cell-penetrating peptides
TNF-α: Tumor necrosis factor
Aβ: Amyloid-β
lncRNA: Long noncoding RNA
DFU: Diabetic foot ulcer
Ang-2: Angiopoietin-2
HUVECs: Human umbilical vein endothelial cells
PI3K: Bisphosphate 3-kinase
TGF-β: Transforming growth factor-β
BMSCs: Bone marrow mesenchymal stem cells
